# Ocean Wind Observation Based on the Mean Square Slope Using a Self-Developed Miniature Wave Buoy

**DOI:** 10.3390/s22197210

**Published:** 2022-09-23

**Authors:** Yao-Zhao Zhong, Hwa Chien, Huan-Meng Chang, Hao-Yuan Cheng

**Affiliations:** 1 College of Physics and Electronic Information Engineering, Minjiang University, Fuzhou 350108, China; 2 Graduate Institute of Hydrological and Oceanic Sciences, National Central University, Taoyuan 320314, Taiwan

**Keywords:** MEMS gyroscope, filtered mean square slope, miniature wave buoy, wind, wave and current

## Abstract

Real-time, continuous, and long-term marine monitoring data benefits ocean research. This study developed a low-cost, multi-parameter, miniature wave buoy. High spatial and temporal resolution of sea surface parameters, including wind, waves, and current, can be obtained at low cost through the deployment of numerous buoys, thus forming an observation array. Tested in the laboratory water tank, the relative error of water surface slope measurement of the buoy was approximately 5.6% when the slope angle was less than 15°. For frequencies between 0.1 and 1.0 Hz, the measurement of slope spectrum was almost identical to that of the wave gauge. The buoy underestimated the slope spectrum between 1.0–1.56 Hz. A good relationship (r^2^ = 0.75) was obtained between wind speed at 10 m above sea surface (U10) and the low-pass-filtered mean square slope (LPMSS). After incorporating the wave age into the U10 inversion process, the root mean square error (RMSE) and BIAS were reduced to 1.15 m/s and 0.02 m/s, respectively. The 2D distribution of buoy-measured slope components was used to detect the wind direction, with an RMSE of 23.7°. The spectral tail slope steepened with increasing wind speed at low wind speeds (<7 m/s). A technical flow chart of the miniature wave buoy is proposed to observe the sea surface parameters. This miniature buoy will play an essential complementary role in the growing demand for sea state monitoring, especially in nearshore oceans.

## 1. Introduction

High-quality marine monitoring data is beneficial to the simulation of ocean processes, marine disaster prediction, offshore navigation safety, and offshore wind power resource assessment. However, sea parameters change dynamically with time and space. Moreover, the analysis of the effects of climate change on the ocean relies on long-term marine data. Thus, monitoring operations need to be intensive, real-time, continuous, and long-term.

In situ data buoys provide continuous long-term reliable and accurate marine data, regardless of the weather and sea conditions, making them a precious source of field measurement data at sea [1]. However, the large size (several meters in diameter and height) and heavy mass (several tons) of traditional data buoys require vessels with sufficient lifting capacity and robust mooring systems for deployment and maintenance [2,3,4]. Besides, the ocean environment is characterized by strong waves and winds, highly saline and humid corrosion, and the core technology of traditional buoys is closed, making the acquisition of data costly. The number and distribution of such data buoys over the sea is therefore minimal. To fill the insufficient space coverage, satellite remote sensing technology for the ocean has developed rapidly. Between 1987 and 2009, the percentage of the total global ocean surface scanned by satellite every six hours rose from 20% to approximately 70% [5]. The Physical Oceanography Distributed Active Archive Center presents the Cross-Calibrated Multi-Platform wind data by combining the results of more than ten satellite wind fields, obtaining higher accuracy of wind speed than that of any single satellite wind field [6]. However, satellite remote sensing data is limited in spatial and temporal resolution and is not available in coastal waters due to the effect of backscatter contamination from land. For example, there were limits due to the fact that the Advanced Scatterometer is not available within 15 km of the shore. As such, a numerical model was adopted to assimilate multi-source data to obtain data with broader coverage than that of in situ observations and a higher time continuity than that of satellite remote sensing. However, the numerical modeling involves the initial boundary condition setting, the nesting method setting, and the process parameter tuning. The simulation process is affected by many factors, and the output data also needs to be examined using a large amount of in situ data.

If the cost of buoys can be reduced, then their deployment in large quantities and areas can be realized; hence, the low spatial coverage of in situ observations at sea will no longer be a problem. An array of observations provides the basis for analyzing the spatial variability of the sea surface parameter fields. The research and development of marine monitoring instruments are moving towards the miniaturization, low cost, diversification of monitoring parameters, and real-time data transmission [7,8,9,10,11,12]. Owing to the rapid development of technology such as microprocessors, it is possible to integrate different functional components into a small chip to form a single system. This study aims to utilize the advantages of the commercial electronics industry and Internet-of-Things technology to integrate sensors and develop a small wave buoy (half-meter-size and a few kilograms in weight). Further, a corresponding algorithm was developed to obtain high precision sea surface parameters such as wind speed and direction, wave height, and wave period.

Moreover, the miniature wave buoy can measure the sea surface Mean Square Slope (MSS) directly, which is vitally important for ocean remote sensing techniques [13]. The MSS is defined as the variance in the sea surface slope and is the basis for almost all satellite remote sensing of sea surface wind [5,14]. Ruf et al. [15] obtained the MSS under the influence of a cyclone by using the Global Navigation Satellite System (GNSS) and then inversed the sea surface wind field, which finally formed the Cyclone GNSS system. The in situ MSS of the sea surface is rarely reported [16]. Moreover, it remains ambiguous how the waves under different growth stages affect the calculated windspeed by influencing the MSS [17]. The wave parameters, which are synchronously measured by the miniature wave buoy, can be further used to analyze the influence of wave state on MSS quantitatively.

In Section 2, we introduce the architecture and algorithms of the self-developed miniature wave buoy. The working principle of the Micro-Electro-Mechanical System (MEMS) gyroscope, which is one of the critical components of the buoy, is described in detail. Section 3 describes the laboratory experiments, including the testing of the water surface slope measurement and frequency response capability evaluation. Section 4 introduces the field observations, presents the construction of the empirical regressions between the wind speed and the MSS, and proposes a method with which to obtain the wind direction based on the two-dimensional distribution function of sea surface slope components. The spectral tail slope is discussed in Section 5 and summarized in Section 6, including the technical flow chart of the miniature wave buoy used to observe the sea surface parameters.

## 2. Instrumentation and Algorithm

### 2.1. Architecture of the Self-Developed Miniature Wave Buoy

The self-developed miniature wave buoy has the shape of a flying saucer. The cross-sections of both the upper and lower hemispheres are shaped to approximate the normal distribution curve, enhancing the fluctuation-following ability of the buoy. The buoy has a height and horizontal diameter of 0.4 m and 0.5 m, respectively, and a mass of approximately 5.0 kg, including the battery. For a detailed description of the structure of the buoy, please refer to Zhong et al. [18].

The microprocessor system is the first core of the miniature wave buoy. This study utilizes a Raspberry Pi Zero processor with 40 GPIO pins, an ARM11 core CPU, a single core with 1 GHz, 512 MB LPDDR2 SDRAM, and a Micro USB2.0 slot. The Raspberry Pi Zero has the lowest power consumption in its class, with 100 mA~1200 mA, and the smallest size of 65 × 30 × 5 mm. The microprocessor system can execute multiple tasks concurrently, including raw data recording, data quality control and calculation, and receiving/sending signals. The raw data consists of pitch, roll, heading, and accelerations in x, y, and z axes with a 10 Hz sampling rate. The data are then used to compute sea surface dynamics parameters such as wave height, wave period, current speed, current direction, wind speed, and wind direction. The computation results are transmitted to the laboratory server database in binary encoded format via the globally covered Iridium satellite communication system. The raw data are recorded on the micro-SD card inside the buoy and can be read if the buoy is retrieved.

The power management module is the second core of the miniature wave buoy. The long-time automatic observation at sea requires high stability and continuity of the power supply. In addition to using low power consumption components, high energy and density batteries as well as solar panels for power supply, this study developed an automatic sleep mode to save power. The buoy consumes approximately 250 mAh during operation and less than 1 mAh in standby, with a typical working voltage is 5.5~18 V. The regular observation process includes system awakening, GPS positioning, 10 Hz raw data observation, online quality control and calculation, and Iridium satellite transmission. Of these, the most power is consumed during data transmission. The buoy can observe around 3600 times in total under the standard condition (alkaline battery and 20 degrees Celsius ambient temperature). Its service life would be 150 days when observing hourly. If the ambient temperature changes to 5 degrees Celsius, the service life will be a half of what it was. The service life can be increased by approximately 4.5 times if the lithium battery is used. In the future, a solar panel is considered to be installed, and the design service life would be around one year.

The third core of the miniature wave buoy is the motion sensor. Benefiting from the development and application of MEMS technology, current marine monitoring equipment such as wave buoys is becoming more miniaturized. Micromotion sensors, depending on their stability and accuracy, can be divided into four grades: commercial, industrial, military, and aerospace grades. Commercial-grade MEMS inertial sensors were adopted because of their low cost and abundant supply in the market. Therefore, the cost of self-developed miniature wave buoy can be greatly reduced to around 1k USD, excluding the labor for assembly and laboratory tests. As for the relatively weak performance of low-cost sensor chips in terms of error, noise, and stability, this study adds filtering and online data quality control to the calculation process.

Furthermore, the GPS signal is used for time correction among the buoys to ensure that the time error between buoys is less than 0.1 s. Moreover, the miniature wave buoy can simultaneously communicate using the radio system to form a spatially networked observation array. This array pattern varies dynamically from time to time and can monitor the ocean on different spatial scales. To be suitable for severe sea conditions, the buoy has a solid structure, and there is no bracket protrusion from the housing (in contrast to conventional buoys that measure wind via an exposed anemometer), which completely avoids damage from heavy wave beating.

### 2.2. Working Principle of the MEMS Gyroscope

The working principle of the MEMS gyroscope is to obtain the angular velocity information by effects such as the Coriolis, levitation or Sagnac effects, and nuclear magnetic resonance. Among them, the MEMS gyroscope designed to use the Coriolis effect is the most common. The Coriolis MEMS gyroscope calculates the angular velocity by measuring the amount of the Coriolis force generated by a moving object. The single-axis oscillation gyroscope is the simplest Coriolis MEMS gyroscope. An internal proof mass is connected to a fixed frame by springs along the *x*- and *y*-axis, which are perpendicular to each other. The *x*-axis is the drive direction and the *y*-axis the sense direction; if the mass is driven with the frequency *ω_x_* in the *x*-axis, the proof mass is made to oscillate with the same frequency, and then the oscillation process is expressed as $x=A_{x}\sin\left(\omega_{x}t\right)$, where *A_x_* is the amplitude and *t* is the time.

According to the principle of Coriolis, there is a Coriolis acceleration *a_y_* on the *y*-axis when the gyroscope rotates around the *z*-axis with the angular velocity Ω*_z_*. The *a_y_* is proportional to the cross product of the moving velocity (*v_x_* = *dx*/*dt*) and the angular velocity (Ω*_z_*), $a_{y}=2\Omega_{z}\times \nu_{x}=2\Omega_{z}A_{x}\omega_{x}\cos\left(\omega_{x}t\right)$, where Ω*_z_* can be calculated by measuring the acceleration or displacement of the mass in the *y*-axis.

There are various ways to measure the small displacement on the *y*-axis. The capacitive type is widely used because of its good compatibility with the integrated circuit manufacturing process and insignificant temperature effect. The self-developed miniature wave buoy chooses the FMS-9 Modules, developed by the CEVA company, as the attitude and acceleration sensors. The performance specifications of FSM-9 are listed in Table 1. In consideration of the power consumption of the system and requirement of ocean observation, the sampling frequency is reduced to 10 Hz.

### 2.3. Algorithms for Data Processing

Phillips [19] proposed a method to obtain the MSS by integrating a wave spectrum as shown in Equation (1),

(1) $$\mathrm{MSS}=\int_{0}^{\infty }k^{2}\varphi \left(\vec{k}\right)d\vec{k}$$

where $\varphi \left(\vec{k}\right)$ is the wave-number spectrum of sea surface waves and $\vec{k}$ is the wave-number vector. By applying the dispersion relationship of the surface wave, the MSS equation is rewritten into frequency spectrum form as shown in Equation (2),

(2) $$\mathrm{MSS}=\int_{0}^{\infty }{\left(\frac{2{\pi f}^{2}}{g}\right)}^{2}F\left(f\right)\mathrm{df}$$

where F(f) is the frequency spectrum of the sea surface elevation. As for the self-developed miniature wave buoy, the two components of the surface slope (pitch and roll) are measured directly. Using the transfer function between S(f) and F(f) derived from linear wave theory, the MSS equation can be estimated using Equation (3):

(3) $$\mathrm{MSS}=\int_{0}^{\infty }S\left(f\right)\mathrm{df}=\int_{0}^{\infty }\left(S_{p}\left(f\right)+{\;S}_{r}\left(f\right)\right)\mathrm{df}$$

where S_p_(f) and S_r_(f) are the frequency spectrum of sea surface pitch and roll, respectively. Before applying Equation (3) to the time series of pitch and roll measured by the buoy, the trends should be removed by subtracting the linear line that best fits the data in the least-squares sense.

The significant wave height (*H_s_*, unit: m) and mean period (*T_m_*, unit: second) are calculated using the following equations: $H_{s}=3.8\sqrt{m_{0}}$ and $T_{m}={\left(m_{0}/m_{2}\right)}^{0.5}$, where *m*_0_ is the zeroth moment of the wave power spectrum, and *m*_2_ is the second-moment wave spectrum. They can be calculated using Equation (4),

(4) $$m_{n}=\int S_{\eta -a}\left(f\right)\cdot f^{n}\mathrm{df}$$

where $S_{\eta -a}\left(f\right)=2\frac{\Delta t}{L}{\left|X_{a}\right|}^{2}\frac{1}{f^{4}}$, and where Δ*t* = sampling interval, *L* = count of data, *X_a_* = Discrete Fourier coefficients of vertical acceleration. The vertical acceleration should be corrected by pitch, roll, and heading.

## 3. Laboratory Calibration

This study uses commercial-grade MEMS motion sensors, which are low-cost and abundant in the market. However, the internal signal processing filter of the sensor is not clear, and so this section is undertaken to check the buoy observation performance, including time domain and frequency domain, in the laboratory water tank.

### 3.1. Time Domain: Accuracy of Water Surface Slope Measurement

**Figure 1:**
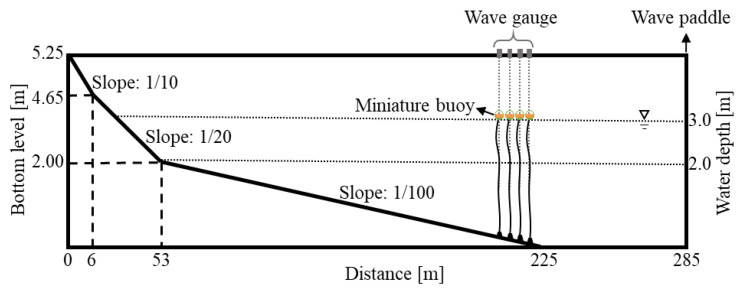
Experiment sketch. The total length of the flume is 300 m. The figure shows only the part until the wavemaker, and the slope in the figure is schematic, not isometric. The capacitive wave gauge is mounted on the sidewall, which records the water level at 25 Hz. The length of the rope for anchoring the miniature wave buoy is 1.5 times the water depth.

The MEMS gyroscope mentioned above is installed within the self-developed miniature wave buoy. A calibration experiment was conducted in a Super Wave Flume with the following dimensions: 300 m × 5 m × 5 m. The root-mean-square errors (RMSE) of the significant wave height, peak period, and mean period are 0.04 m, 0.02 s, and 0.14 s, respectively. The layout of the calibration experiment is illustrated in Figure 1. The horizontal axis shows the distance from the end of the tank. Four miniature buoys were anchored near 225 m, the distance between two adjacent buoys is 3 m, and the wavemaker is located at 285 m. A capacitive wave gauge was set on the wall of the tank at each anchor point of the buoy to record the water level with a sampling frequency of 25 Hz. The structure of the gauge is simple, and the disturbance of the wave surface negligible. The floating rope is used for anchoring to avoid additional drag of the rope. The length of the rope is 1.5 times the water depth, giving the buoy sufficient space for orbital motion with the waves. The wavemaker produces 12 different sets of regular waves, namely (period, wave height): (3.0 s, 0.2 m|0.4 m|0.5 m|0.6 m|0.8 m), (5.0 s, 0.4 m|0.5 m|0.6 m|0.8 m), and (7.0 s, 0.4 m|0.5 m|0.6 m). As each group continues for 10 min, the buoy measures continuously from the second minute to the ninth minute. After each group wave, the water tank rests for 10 min to obtain the fully calm water surface so that the residual waves cannot affect the next group experiment.

**Figure 2:**
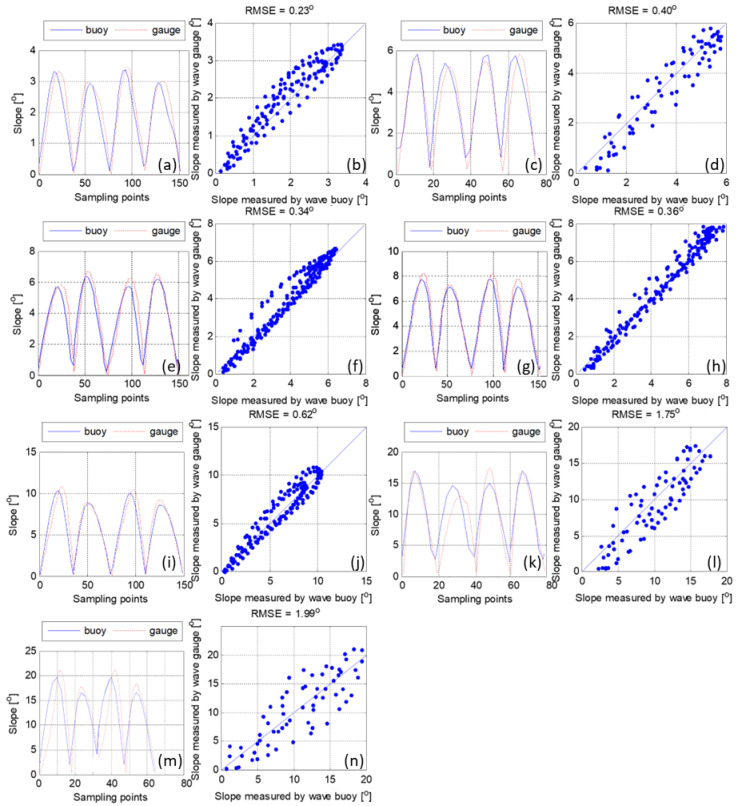
Surface slope measured using wave buoy data (solid blue line) compared to the water elevation gradient calculated using wave gauge data (red dashed line) under different wave steepness. Since waves with different periods and heights may produce similar slope, panels (**a**–**n**) are the results from selected experiment cases with different wave parameters settings.

A comparison of the water surface slope calculated from the pitch, roll, and yaw, measured by the miniature buoy and that was calculated from the wave gauge is shown in Figure 2. The horizontal angle is defined as zero degrees, and all the inclination angles are positive degrees in the figure. The 12 groups of waves with different periods and heights produce water surface slopes ranging from 0 to 20°. The results show that, the greater the angle of water surface slope, the larger the RMSE: the RMSE is approximately 0.23° when the slope angle is below 5°; the RMSE is approximately 0.36° when the slope angle ranges from 5–8° (Figure 2c–h); the RMSE is approximately 0.62° when the slope angle is approximately 10° (Figure 2i,j); the RMSE increases to 1.75° when the water slope angle reaches about 17° (Figure 2k,l), and the highest RMSE is approximately 1.99° when the water surface slope angle reaches 20° (Figure 2m,n). The average relative error is approximately 5.6% when the water surface slope angle is less than 15° and increases rapidly to approximately 9.4% for the water surface slope angle over 15°. Besides, when the water surface slope angle is less than 15°, the angle measured by the miniature buoy remains 0–1° at the wave crest or trough. If the water surface slope angle exceeds 15°, the measurement error of the miniature buoy at the wave crest or trough enlarges to approximately 2–3°. This is probably because of the insufficient response to the high-frequency variation of the water surface due to the miniature inertia of the buoy (discussed in detail in the next section). It can be concluded that the miniature buoy can accurately measure the water surface slope by using the built-in MEMS gyroscope.

### 3.2. Frequency Domain: Frequency Response Curve of the Miniature Wave Buoy

Due to the inertia, the wave-following property of the miniature buoy is not ideal, meaning that the buoy cannot adequately respond to the high-frequency fluctuation of the sea surface in time. Meanwhile, the noise-of-slope measurement becomes relatively high when the surface slope is small. This phenomenon is more significant for longer wavelength or smaller slope angle. In this section, the frequency response characteristics of the miniature buoy are analyzed.

**Figure 3:**
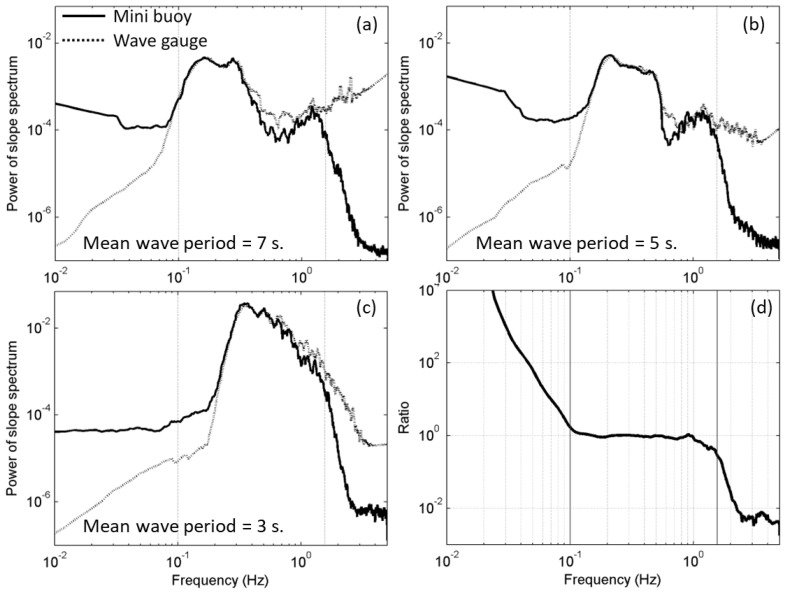
Slope spectrum measured using the miniature buoy (solid lines) and wave gauge (dotted lines) under the wave conditions with mean wave period of 7 s (**a**), 5 s (**b**), and 3 s (**c**). The ratio of the slope spectrum measured using the miniature wave buoy to the slope spectrum measured by the wave gauge (**d**). Each panel is marked with two vertical solid lines at 0.1 Hz and 1.56 Hz, which are the upper and lower cutoff frequency of the BPMSS.

In the current study, the 25 Hz water level recorded was used as a criterion to evaluate the frequency response capability of the buoy. Before comparison, the wave-gauge-measured F(f) is transferred to wave-gauge-measured S(f) (dotted lines in Figure 3a–c) by using the transfer function derived from linear wave theory. The solid black lines in Figure 3a–c represent the buoy-measured S(f). The results show that, for different mean wave periods, the frequency bands in which the buoy-measures S(f) agree with the wave-gauged-measured S(f) differ. For a mean wave period of 7 s (Figure 3a), the consistent frequency band is approximately 0.08–1.51 Hz; for the 5 s (Figure 3b), the consistent frequency band is approximately 0.15–1.56 Hz; and for the 3 s (Figure 3c), the consistent frequency band is approximately 0.21–1.56 Hz. The frequency response capability of the miniature buoy may seem to vary with the wave period, but this is not actually the case, because the wavemaker can only produce one kind of wave in a single experiment. To obtain a comprehensive view of the frequency response capability of the buoy, the ratios are integrated into Figure 3d. As shown, for frequencies less than 0.1 Hz, the miniature wave buoy can significantly overestimate; for frequencies between 0.1 and 1.0 Hz, the measurement is almost identical to that of the wave gauge. At frequencies above 1.0 Hz, the higher the frequency, the more the measurement of the buoy is underestimated. It should be noted that the wave period in the experiment ranges from 3 to 7 s, and the wave height ranges from 0.2 to 0.8 m due to the performance limitation of the wavemaker. If other waves can be produced, the frequency range that the miniature wave buoy can accurately measure should be extended wider.

However, even under ideal wavemaking conditions, there is always a limit to the frequency range in which the slope ratio equals one. When calculating MSS, the ocean surface low-pass-filtered mean square slope with an upper bound is frequently used (as shown in Equation (5)):

(5) $$\mathrm{LPMSS}=\int_{0}^{\mathrm{ufc}}S\left(f\right)\mathrm{df}$$

where ufc is the upper cutoff frequency, which usually ranges from one-sixth to one-third of the working wavenumber of the altimeter (e.g., [20,21,22,23]). The first systematic LPMSS data set is reported by Cox and Munk [20]. In this study, the upper bound cutoff frequency is determined by the combined frequency response capability of the miniature buoy and operating frequency band of the GNSS-Reflectometry. The GNSS-Reflectometry is a promising remote sensing technology whose operation mode is equivalent to a multi-base L-band radar system with artificial radiation source, transceiver split, and multiple transceivers. Thus, it possesses the advantages of both active and passive remote sensing, which is gaining more attention (e.g., [24,25,26,27,28]). The corresponding electromagnetic wavenumber of the L band frequency (1.41 GHz) is 29.53 rad m^−1^. The upper bound of the MSS integration is taken as one-third of the electromagnetic wave number [22,23], and the corresponding ufc is 1.56 Hz based on the deep water–wave dispersion relationship. Two vertical solid lines are shown in Figure 3d, with the right one marking the position of 1.56 Hz. As shown, the measurements of the buoy in the 1.0–1.56 Hz frequency range are slightly underestimated, but still in the same order of magnitude. This frequency range corresponds to a wavelength of 0.64–1.56 m based on the relationship between wavelength and period in deep water (L = 1.56T^2^). The diameter of the buoy is 0.5 m. The underestimation of the buoy in this frequency range should be related to its size. Moreover, the higher the frequency in Figure 3d above 1.0 Hz, the more underestimated were the measurements of the buoy. Newman and Landweber [29] indicated that the frequency response coefficient (K) of a floating object on the sea surface is related to its own natural frequency and damping coefficient: $K=\frac{1}{\sqrt{{\left(1-{\;\Lambda }^{2}\right)}^{2}+4\mu^{2}\Lambda^{2}}}$, where Λ is the ratio of water wave frequency and the buoy’s natural frequency, and μ is the ratio of the buoy’s damping coefficient and the buoy’s natural frequency. The natural frequency of the self-made buoy in this study was calibrated with the laboratory water tank and was approximately 1.0 Hz. According to the above equation, the K decreases rapidly with increasing frequency. This is consistent with the frequency response characteristics of the buoy observed in the water tank experiment (Figure 3d). The vertical line on the left in Figure 3d marks the position of 0.1 Hz. Fluctuations below 0.1 Hz frequency are significantly overestimated. Accordingly, 0.1 Hz is used as the lower bound cutoff frequency (lfc) and Equation (5) is adjusted to Equation (6):

(6) $$\mathrm{BPMSS}=\int_{\mathrm{lfc}}^{\mathrm{ufc}}S\left(f\right)\mathrm{df}=\int_{\mathrm{lfc}}^{\mathrm{ufc}}\left(S_{p}\left(f\right)+{\;S}_{r}\left(f\right)\right)\mathrm{df}$$

where BPMSS is the ocean surface band-pass-filtered mean square slope.

**Figure 4:**
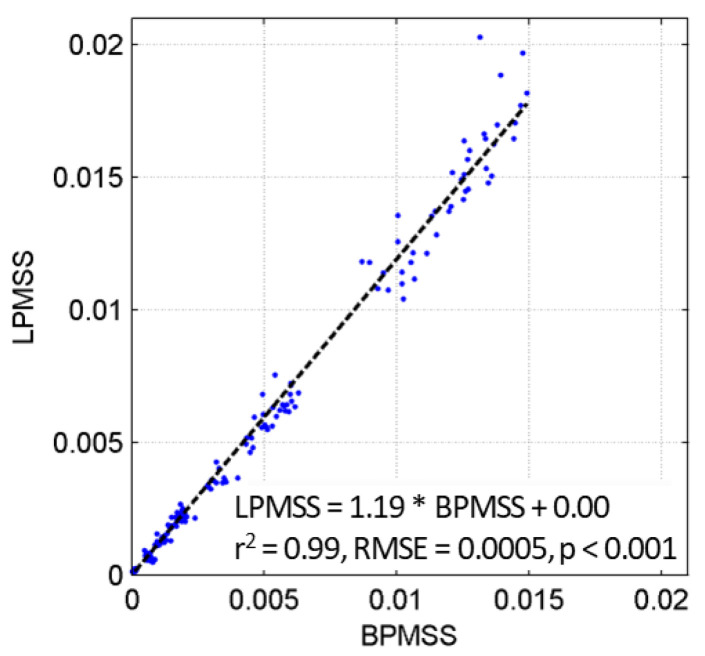
Empirical relationship between BPMSS and LPMSS. The BPMSS is calculated by applying Equation (6) to the slope spectrum measured by the miniature wave buoy, and the LPMSS is calculated with Equation (5) to the slope spectrum measured by the wave gauge.

The BPMSS does not consider fluctuations whose frequencies are below 0.1 Hz and underestimates the contribution of fluctuations in the range of 1.0–1.56 Hz. Thus, the BPMSS is corrected to LPMSS by the empirical relationship shown in Figure 4. The horizontal axis of Figure 4 is the BPMSS calculated by applying Equation (6) to the slope spectrum measured by the miniature wave buoy, and the vertical axis is the LPMSS calculated applying Equation (5) to the slope spectrum measured by the wave gauge. The results are significantly correlated: LPMSS = 1.19 ∗ BPMSS + 0.00 (r^2^ = 0.99, *p* < 0.001). Accordingly, Equation (6) is redefined to Equation (7) as:

(7) $$\mathrm{LPMSS}=1.19\int_{\mathrm{lfc}}^{\mathrm{ufc}}\left(S_{p}\left(f\right)+{\;S}_{r}\left(f\right)\right)\mathrm{df}$$

The slope spectrum measured by the buoy decreases rapidly from approximately 1.5 Hz (solid lines in Figure 3a–c). The decay trend, on average, is approximately about f^−8^, which is steeper than Kolmogorov’s −5/3 Law. The miniature buoy is a highly effective low-pass filter due to its inertia and size.

## 4. Field Observations

### 4.1. Regression Analysis between U10 and In Situ LPMSS

In situ measurements of LPMSS are challenging. The first such measurement was recorded by Cox and Munk [20], who used photographs of sun glitter taken from an aircraft to estimate sea surface roughness. Yan et al. [30], in their analysis of sea surface slope distribution affecting Ku-band precipitation radar measurements, obtained the required LPMSS dataset by integrating spectral models. Li et al. [17] used the wave spectra measured by the National Data Buoy Center at the sea surface to calculate the LPMSS; however, the upper cutoff frequencies of the buoy were in the 0.4–0.5 Hz range; thus, it was not possible to observe the MSS caused by wind waves. Compared with previous studies, the miniature wave buoy developed here has three advantages: (1) the direct observation avoids the tedious calculation steps of remote sensing and also avoids the idealized assumptions of the ideal spectra model; (2) the upper frequency response limit has been increased to approximately 1.56 Hz, providing a complete frequency coverage of wind waves; (3) it is easier to obtain accurate LPMSS results based on the surface slope spectrum than based on the surface elevation spectrum [22].

A field experiment using a miniature buoy was conducted near the mouth of the semi-enclosed Baisha Bay, in northern Taiwan, with the mouth opening to the north-northwest. The deployment style was the same as that in the water tank experiment. A floating rope 1.5 times the water depth was used to connect the miniature buoy with the heavy blocks on the bottom bed, providing the buoy with the space to float freely with the water surface and at the same time ensuring that the buoy stayed within Baisha Bay throughout the observation. The wind speed and direction data for comparison were obtained from a meteorological station on the shore of Baisha Bay, approximately 500 m away from the anchored buoy. The synchronous observation was conducted from 20 January to 20 March 2019, for a total of 60 days.

**Figure 5:**
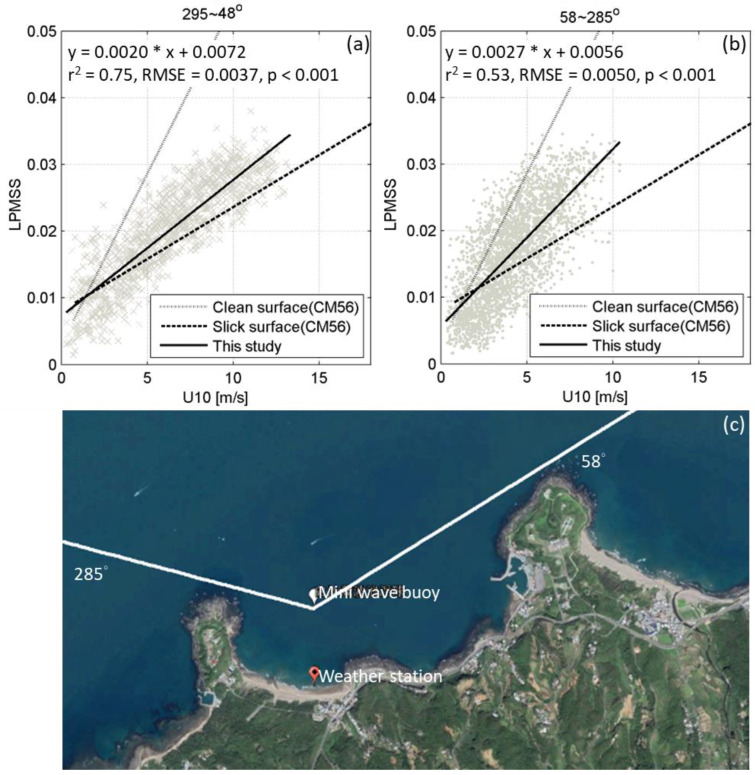
Relationship between U10 and LPMSS measured by the miniature buoy. (**a**) Onshore and (**b**) offshore wind conditions. (**c**) Criteria for distinguishing whether wind conditions are influenced by coastal topography.

The growth of the wind wave was controlled by three factors: wind speed, wind fetch, and wind duration. If the wind fetch is large and wind duration long enough, then a good relationship can be obtained between the wind and wave parameters. However, if the wind fetches are limited or the wind duration changes, the relationship will differ. This is because the full transmission of wind speed information to the sea surface fluctuations depends on sufficient blowing time and space. There will also be a significant difference in LPMSS between winds affected by coastal sheltering and those not sheltered. Figure 5 shows the correlation between U10 and LPMSS. The U10 is divided into two groups according to whether the wind is sheltered by coastal topography, corresponding to Figure 5a (unshaded) and Figure 5b (sheltered). The steps for dividing the U10 are as follows: (1) measuring the orientation of lines between each cape of Baisha Bay and the anchored miniature buoy. The orientation of the western line is 285° (with north as 0°, increasing clockwise, the same below), and the eastern orientation is 58° (Figure 5c); (2) the wind direction in the range of 58–285° is classified as coast-sheltered wind; (3) since the topography of the cape may still affect the wind sweeping over the cape, the direction range in which the wind is not sheltered by the coast is reduced from 285–0–58° to 295–0–48°.

Figure 5a shows the result for when the wind is not sheltered by the coast: LPMSS = 0.0020 ∗ U10 + 0.0072 (r^2^ = 0.75, RMSE = 0.0037, *p* < 0.001). Figure 5b illustrates the result for when the wind is sheltered by the shore: LPMSS = 0.0027 ∗ U10 + 0.0056 (r^2^ = 0.53, RMSE = 0.0050, *p* < 0.001). The correlation coefficient of the latter is significantly lower because of the limitation of wind fetch due to the influence of land. The dotted and dashed lines in Figure 5a,b show the relationship between U10 and LPMSS estimated by Cox and Munk [31] based on the intensity and area of the sea surface–sun glitter at different wind speeds on a clean and slick surface, respectively. The LPMSS observed by the miniature buoy is almost always smaller than the results of the clean surface (except for U10 less than 2 m/s). This is reasonable, because the LPMSS calculated through the sun glitter on the sea surface is almost a complete integration of frequency. Simultaneously, the miniature buoy has no response to the high-frequency fluctuation due to its mass and size.

### 4.2. Effect of Wave Age on in situ LPMSS

**Figure 6:**
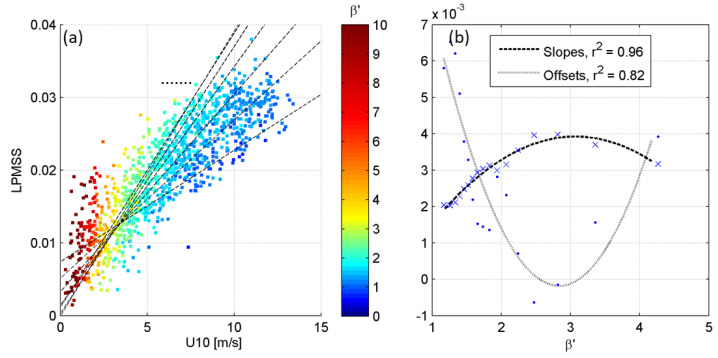
(**a**) Illustration of the grouped linear fittings according to the *β*′. (**b**) The corresponding slopes and intercepts of the fitted lines for all subgroups.

**Figure 7:**
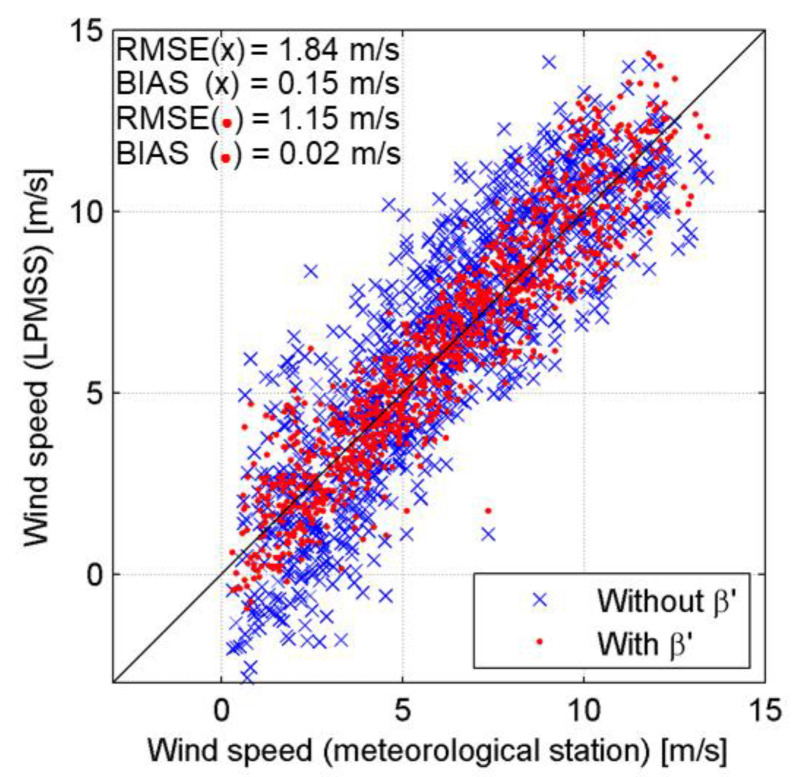
Comparison of wind speed derived from methods with or without wave age (*β*′). The horizontal axis of is the U10 measured by the meteorological station, and the vertical axis is the U10 inferred from LPMSS. The blue crosses and red dots indicate the results without and considering *β*′, respectively.

During the development of wind waves, high-frequency component waves will transfer energy to low-frequency ones through nonlinear interactions. With the accumulation of wave energy in the low-frequency part of the wave energy spectrum, the sea surface wave height and wavelength will gradually increase, together with the wave phase speed. In other words, when the wave development stage changes, even for the same wind speed conditions, the corresponding sea surface roughness will also change. The wind waves at different stages of growth can be denoted by the wave age, which is traditionally defined as the ratio of wave phase speed to wind speed. Therefore, the wave age is a critical factor in distinguishing the wind wave and the swell [32]. However, because the wave speed is the to-be-determined parameter, the pseudo-wave age (*β*′) is used instead for the wind speed inversion through LPMSS [17,33], as per Equation (8):

(8) $$\beta^{’}=3.24{\left(\frac{gH_{s}}{U_{10}^{2}}\right)}^{0.62}$$

where *g* is the gravity acceleration, *H_s_* is the significant wave height, and U10 is the wind speed at 10 m above the sea surface. The relationship between LPMSS and U10 varies for different wave stages. This study constructed the relationships based on the first 30 days of observational data (Figure 6) and is applied with the second 30 days of data (Figure 7).

Figure 6a presents an illustration of the grouped linear fittings. The slope of the regression equation increases with increasing wave age. An increase in slope means that the contribution of U10 to LPMSS becomes higher, which reflects that the larger the wave age, the more energy is transferred to the wave from the wind. Thus, the same LPMSS corresponds to a significantly different U10 as the wave age varies. For instance, with LPMSS = 0.02, the corresponding U10 ranges from 2–10 m/s. The difference in U10 is approximately 5-fold. This difference becomes more prominent as U10 increases, revealing that the regression equations without differentiating the wave ages will significantly produce errors in the wind speed estimation. For a detailed understanding of the variation characteristics of the relationship between LPMSS and U10 at various wave stages, this study performed a grouping fitting according to the *β*′ value. In particular, partial overlap of data was made between adjacent groups to ensure that the sample size of each group remained statistically significant when the data was split into many groups. The corresponding slopes and intercepts of the fitted lines for all subgroups are plotted in Figure 6b. The correlation coefficients of these linear fits are 0.83 ± 0.03 (mean ± standard deviation), and all the regression results were statistically significant (*p* < 0.001). From the figure, as *β*′ increases, the slope first increases and then decreases, and the intercept decreases at first and then increases. The slopes and intercepts were fitted with quadratic curves, and the high values of r^2^ were obtained as 0.96 and 0.82, respectively.

To examine the improvement of the U10 inversion results considering *β*′, the empirical relationship described above was applied to the LPMSS data observed in the latter 30 days. The steps are as follows: (1) *β*′ was calculated with an initial value of U10′; (2) the corresponding slope and intercept from the empirical relations in Figure 6b were calculated; (3) LPMSS was converted to U10″ based on the slope and intercept; (4) if the U10″ was smaller (larger) than U10′, then U10′ increased (decreased) as the new initial value; (5) the above steps were continued until the difference between U10′ and U10″ was reduced to an acceptable level. The results after applying the method are shown in Figure 7. The horizontal axis is the U10 measured by the meteorological station, and the vertical axis is the U10 inferred from LPMSS. The blue crosses and red dots indicate the results without and considering *β*′, respectively. The distribution of the red dots is more concentrated than that of the blue crosses, revealing a significant improvement in the quality of the results obtained by considering the wave age for the U10 inversion. Specifically, the RMSE reduced greatly from 1.84 m/s to 1.15 m/s, and the BIAS reduced from 0.15 m/s to 0.02 m/s after considering the wave age. Sousa et al. [34] conducted a comparison analysis between sea surface winds obtained from the Quick Scatterometer and in situ observations from the oceanographic buoys moored in the coastal ocean of Galicia, Spain. The accuracy of the wind speed derived from the Quick Scatterometer varied between 1.5 and 2.0 m/s (RMSE), and the calculated BIAS was 0.5 m/s. Wang et al. [35] reported that the RMSE and BIAS of wind speed derived from the HY-2B Scatterometer were 0.98~1.22 m/s and 0.07~0.32 m/s, respectively, by comparison with the buoy measurements. Remmers et al. [36] estimated the performance of the Advanced Scatterometer in measuring the wind speed, which slightly overestimated the in situ data by 0.09 m/s (BIAS) with the RMSE of 0.90 m/s. In brief, the miniature wave buoy developed in this study achieved a good accuracy in wind speed inversion by LPMSS, although the maximum wind speed during the field observation was only 14 m/s. It is unclear whether the established empirical correlation equation is applicable to wind speeds above 14 m/s; therefore, the performance of the wave buoy under high wind speed needs to be further evaluated.

It is worth noting that there is no available satellite wind speed in nearshore waters due to the terrestrial influence on the backscatter. Moreover, because of the high cost of traditional meteorological data buoys, the availability of these buoys in nearshore waters is minimal. The self-developed miniature wave buoy has apparent advantages in cost and spatial-temporal resolution in providing wind/wave/current field data in nearshore waters. It will improve the monitoring of sea conditions in nearshore waters.

### 4.3. Detection of Mean Wind Direction Based on the 2D Distribution of Slope Components

**Figure 8:**
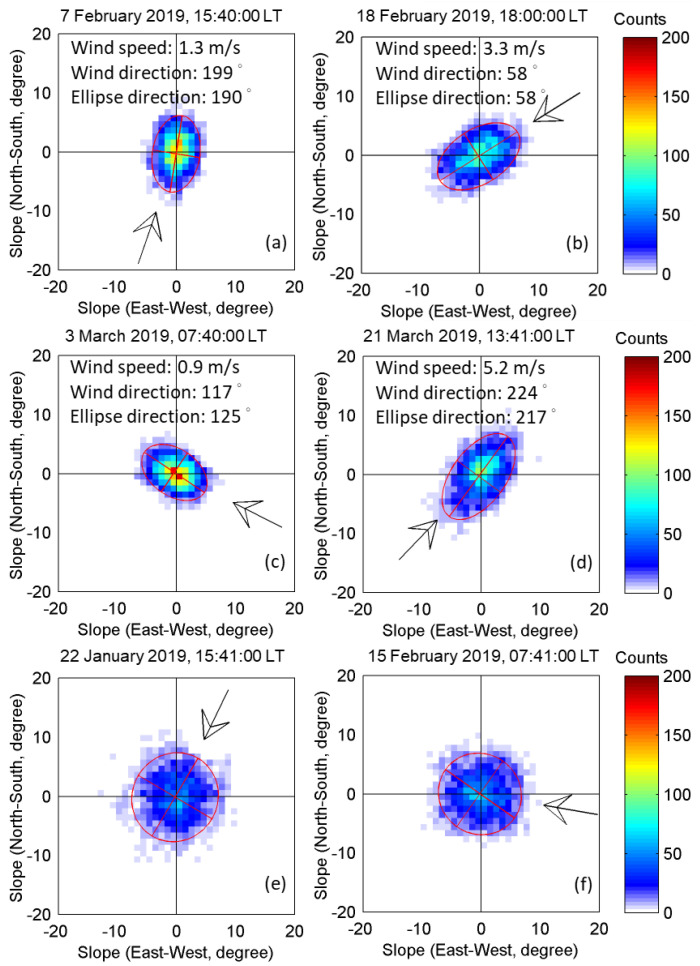
2D probability distribution of sea surface slope components under different wind speeds and directions (**a**–**d**). The cases that the wind direction cannot be detected (**e**,**f**). The *x*-axis represents the slope in the east–west direction, and the *y*-axis represents the slope in the north–south direction. The long and short axis of the best-fit ellipse are shown as red lines. The arrow shows the wind direction measured by the meteorological station at the coast.

In the linear wave theory, the sea surface slope is assumed to conform to a Gaussian distribution. Cox and Munk [20,37] showed that the actual sea surface slope is more consistent with the Gram–Charlier distribution. Compared to the Gaussian distribution, the Gram–Charlier distribution has two additional factors: peakedness and skewness. Fung and Chen [38] introduced the surface correlation function to describe the statistical correlation of the sea surface height at any two points in a given direction. They analyzed the correlation function of the multi-scale rough surface, which presented exponential variation patterns. Ulaby and Long [39] further noted that the total surface correlation function on a given direction *φ* was a bi-exponential function with a symmetric center origin. The correlation length along *φ* direction can be defined as *L*_total_ = *L*_upwind_ ∗ cos^2^*φ* + *L*_crosswind_ ∗ sin^2^*φ*, where *L*_upwind_ stands for the surface correlation length in upwind direction (*φ* = 0), while *L*_crosswind_ stands for the correlation length in crosswind direction (*φ* = 90). As the *L*_crosswind_ is expected to be longer than *L*_upwind_, and both of them are decreasing functions of wind speed [39], the horizontal cross-section of the total surface correlation functions is an ellipse shape. As shown in Figure 8, the two-dimensional probability distribution of the sea surface slope components is in fact an ellipse shape. The original records of pitch, roll, and heading were converted to the surface slope components *Slope*_East-West_ and *Slope*_North-South_ using Equation (9). Applying these properties of the surface correlation function, it can be deduced that the long axes in Figure 8 refer to the upwind direction, while the short axes refer to the crosswind direction.

(9) $$Slope_{\mathrm{East}-\mathrm{West}}=\frac{\sin\left(heading\right)\sin\left(pitch\right)}{\cos\left(pitch\right)}+\frac{\cos\left(heading\right)\sin\left(roll\right)}{\cos\left(roll\right)}\;Slope_{\mathrm{North}-\mathrm{South}}=\frac{\cos\left(heading\right)\sin\left(pitch\right)}{\cos\left(pitch\right)}-\frac{\sin\left(heading\right)\sin\left(roll\right)}{\cos\left(roll\right)}$$

Depending on the direction of the best-fit ellipses of the long axis, the mean wind direction is restricted to only two directions that differ by 180°. As shown in Figure 8a, the upwind direction is 10° or 190°, while the mean wind direction observed at the nearby meteorological station, expressed as an arrow (hereinafter), is 199°. Figure 8b has the potential upwind direction of 58° or 238°, and the actual wind direction is 58°. Figure 8c has the potential upwind direction of 125° or 305°, and the actual wind direction is 117°. Figure 8d has the potential upwind direction of 37° or 217°, and the actual wind direction is 224°. Still, no significant differences between the elliptical long and short axes in some periods were found. Thus, the wind direction could not be estimated (e.g., Figure 8e,f), which should be due to the unstable wind direction during the observation period. In this study, the wind direction could not be detected when the ratio of elliptical long and short axis length was less than 1.1.

**Figure 9:**
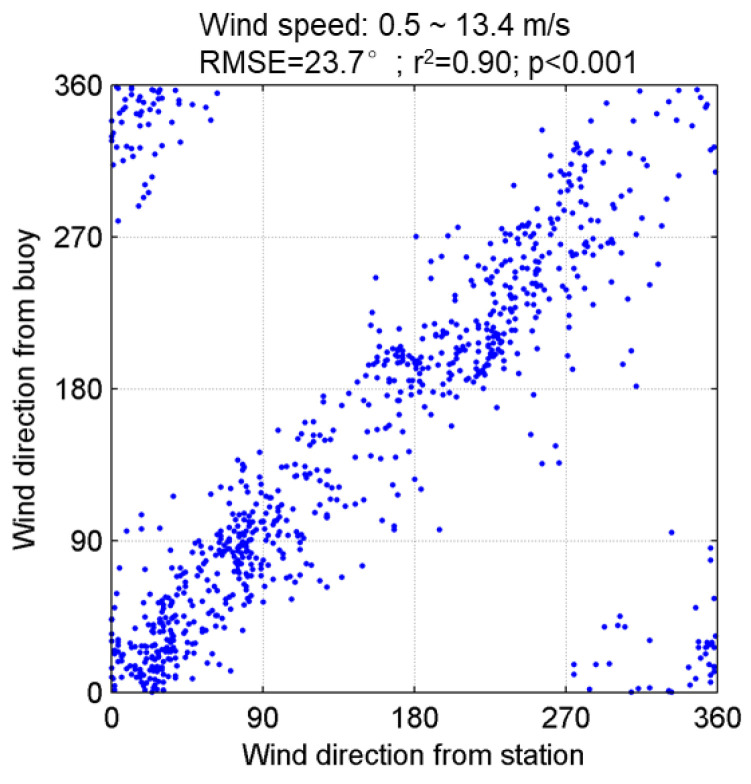
The wind direction comparison between miniature wave buoy and meteorological station.

For the observation data with ratios greater than 1.1, the wind direction measured with the meteorological station was used to choose the ultimate wind direction from two potential wind directions evaluated by the miniature wave buoy. The wind direction comparison between the buoy and the meteorological station is shown in Figure 9. The RMSE is 23.7° (r^2^ = 0.9, *p* < 0.001), indicating that the wind direction measurement of the miniature wave buoy is reliable. The accuracy of the miniature wave buoy in observing wind direction is better than that of Quick Scatterometer, whose RMSE is approximately 35° [34], and is comparable to that of the HY-2B Scatterometer. Wang et al. [35] reported that the RMSE of wind direction derived from the HY-2B was 19.63~25.69° by comparing with buoy measurements.

## 5. Discussion: Spectral Tail Slope Analysis

**Figure 10:**
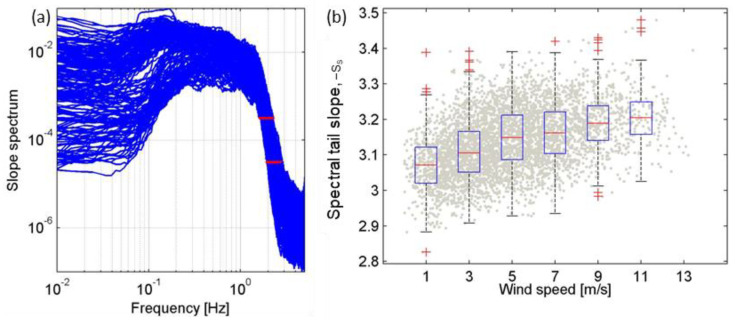
(**a**) The frequency spectrum of sea surface slope measured by miniature wave buoy during the field observation. (**b**) Boxplot of spectral tail slopes (ss) under different wind speed conditions.

First, the following spectral tail slopes must be clarified: tail slope of the frequency spectrum of sea surface slope S(f), denoted as ss; and that of the frequency spectrum of sea surface level F(f), denoted as sf. According to the linear wave theory, we can deduce that ss = sf + 4. The spectral tail slope is a key parameter of the equilibrium spectrum, which was first adopted by Phillips [40] to analyze short-scale waves. Many theoretical equilibrium spectral models use a constant sf between −4 and −5 [40,41,42,43]. However, the range of sf observed in the field is much wider, such as the directional wave spectra through buoys [44], and wave spectra through wire gauges on a buoy [45], both of which yielded sf as steep as −7. Similar results regarding sf were found in the present study. In practice, the spectrum values between 1.5fp and 3fp [46] or 2fp and 4fp [44] were fitted to obtain the sf, where fp was the peak frequency. The S(f) measured in this study is shown in Figure 10a. The two red lines in the figure are marked 10^−3.5^ and 10^−4.5^ correspond to the central region of 2fp to 4fp, respectively; hence, the definition by Young [44] is more applicable to this study. Hwang [22] calculated sf using the definition by Young [44] and conducted a probability distribution function analysis of sf for three different U10 ranges (7–10 m/s, 10–15 m/s, and >15 m/s), and although only three sets of data were available, they showed that, the higher the wind speed, the steeper the spectral tail slope. This study created more subgroups, presented as a boxplot in Figure 10b. The red line indicates the median of each group, while the upper (lower) boundary of the blue rectangle represents the 75% (25%) quantile of each group. The distance between the upper and lower boundaries of the rectangle is defined as the interquartile distance, and the length of the black dashed line extending from the rectangle is 1.5 times the interquartile distance, or up to the maximum/minimum value of the group data, whichever is shorter. If the data are distributed outside the black dashed line (approximately 99.3% coverage if the data are normally distributed), it is judged as an outlier and marked with a red plus symbol. The spectral tail slope steepens with increasing wind speed even at low wind speeds (<7 m/s). This is important supplementary to the probability distribution function analysis by Hwang [22] who also suggested that the LPMSS is less sensitive to the spectral tail slope when U10 is less than 10 m/s. However, there are five data sets (Figure 10b) with U10 smaller than 10 m/s in this study, thus making it possible to quantify the lower sensitivity: for every 1 m/s increase in U10, -ss increases by 0.015.

## 6. Conclusions

This study takes advantage of the current developments and trends in the consumer electronics industry and Internet-of-Things technology to develop a miniaturized marine monitoring buoy that integrates MEMS gyroscopes, global positioning chips, and other sensors on an Mirco-Control-Unit platform, which reduces the size, weight, and cost of the buoy. After the laboratory water tank calibration and field testing, the accuracies of the self-developed miniature wave buoy in measuring wind speed and direction were 1.15 m/s and 23.7°, respectively.

**Figure 11:**
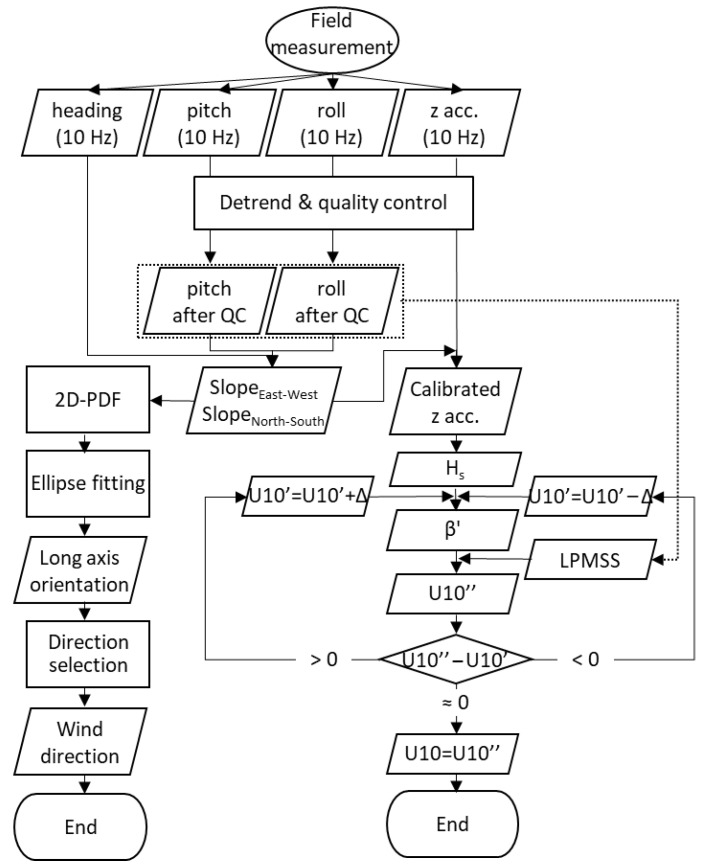
Workflow of ocean wind observation using the miniature wave buoy. The “acc.” is short for acceleration. The QC is an abbreviation for quality control. The PDF is an abbreviation for probability distribution function. Δ represents a small value, such as 0.1 m/s.

A workflow of the miniature wave buoy is summarized in Figure 11. The specific processes are as follows: (1) attitude parameters and tri-axis accelerations are measured through MEMS gyroscope with 10 Hz sampling frequency; (2) LPMSS, *Slope*_Ease-West_, and *Slope*_North-South_ are calculated based on the attitude parameters after quality control; (3) the 2D probability distribution function of *Slope*_Ease-West_ and *Slope*_North-South_ are plotted and the ellipse fitting is carried to obtain the direction of the long axis; (4) U10 is solved iteratively based on the empirical relationship between LPMSS, U10, and *β*’.

Compared to traditional meteorological data buoys, the miniature wave buoy has a simpler and more compact structure, smaller size, and lighter mass. Its deployment and mooring are straightforward to realize. Benefit from the lower manufacturing costs, the miniature wave buoy is better suited for deployment in larger numbers to achieve ample space coverage. More importantly, due to the reduced size and mass of the miniature wave buoy, its motion inertia also becomes smaller. It can respond to a higher frequency and shorter wavelength of sea surface fluctuations and has better sea surface following ability. Compared with satellite wind data, the self-developed miniature wave buoy also has obvious spatial and temporal resolution advantages. The proposed miniature wave buoy will play an essential role in the growing demand for nearshore sea state monitoring.

## Figures and Tables

**Table 1:** Performance specifications of FMS-9 Modules developed by CEVA company.

No.	Item	Detail
1	Attitude parameters	pitch, roll, heading
2	Acceleration parameters	x-, y-, z-acceleration, right-hand coordinate system
3	Range of angular velocity	+/−1833°/s
4	Resolution of angular velocity	0.05°/s
5	Range of linear acceleration	+/−4 g
6	Resolution of linear acceleration	6 mg
7	Sampling rate	125 Hz

## Data Availability

All data, models, or code generated or used during the study are available from the corresponding author by request.
